# The Potential of CRISPR/Cas Technology to Enhance Crop Performance on Adverse Soil Conditions

**DOI:** 10.3390/plants12091892

**Published:** 2023-05-05

**Authors:** Humberto A. Gajardo, Olman Gómez-Espinoza, Pedro Boscariol Ferreira, Helaine Carrer, León A. Bravo

**Affiliations:** 1 Laboratorio de Fisiología y Biología Molecular Vegetal, Instituto de Agroindustria, Departamento de Ciencias Agronómicas y Recursos Naturales, Facultad de Ciencias Agropecuarias y Medioambiente & Center of Plant, Soil Interaction and Natural Resources Biotechnology, Scientific and Technological Bioresource Nucleus, Universidad de La Frontera, Temuco 1145, Chile; h.gajardo01@ufromail.cl (H.A.G.); olmang03@gmail.com (O.G.-E.); 2 Centro de Investigación en Biotecnología, Escuela de Biología, Instituto Tecnológico de Costa Rica, Cartago 30101, Costa Rica; 3 Department of Biological Sciences, Luiz de Queiroz College of Agriculture (ESALQ), University of São Paulo, Piracicaba 13418-900, Brazil; boscariolfp@gmail.com (P.B.F.); hecarrer@usp.br (H.C.)

**Keywords:** genetic engineering, extremophytes, food security, abiotic stress

## Abstract

Worldwide food security is under threat in the actual scenery of global climate change because the major staple food crops are not adapted to hostile climatic and soil conditions. Significant efforts have been performed to maintain the actual yield of crops, using traditional breeding and innovative molecular techniques to assist them. However, additional strategies are necessary to achieve the future food demand. Clustered regularly interspaced short palindromic repeat/CRISPR-associated protein (CRISPR/Cas) technology, as well as its variants, have emerged as alternatives to transgenic plant breeding. This novelty has helped to accelerate the necessary modifications in major crops to confront the impact of abiotic stress on agriculture systems. This review summarizes the current advances in CRISPR/Cas applications in crops to deal with the main hostile soil conditions, such as drought, flooding and waterlogging, salinity, heavy metals, and nutrient deficiencies. In addition, the potential of extremophytes as a reservoir of new molecular mechanisms for abiotic stress tolerance, as well as their orthologue identification and edition in crops, is shown. Moreover, the future challenges and prospects related to CRISPR/Cas technology issues, legal regulations, and customer acceptance will be discussed.

## 1. Introduction

Humans have depended on plants throughout their existence. Since the beginning of agriculture and the domestication of plants, agronomic management and traditional breeding have provided humanity with the modern varieties that feed the world today [1]. In contrast to the primary evolution of land plants, which occurred under unfavorable conditions (e.g., drought, fluctuating light, and temperature) [2], domestication occurred under relatively stress-free, managed conditions [3,4]. Later, during the green revolution of the mid-twentieth century, agricultural breeding radically modified plant architecture to achieve high yields [5]. As a result, the current world situation is that crop plants are much more used as food and feed than wild species [6].

Unfortunately, improvement of yield-related traits can compromise resource allocation to other traits, impairing biotic and abiotic stress tolerance [4]. This trade-off between traits hinders the capacity of crop species to mitigate the effect of changing environmental conditions [7]. Therefore, crop domestication increased the likelihood of these species being more sensitive to stresses than their wild relatives [8,9]. Within this framework, some crops can only achieve high yields with management-intense modern agricultural practices [10].

Indeed, crop production is already being affected across several regions worldwide due to climate change. The rising frequency of extreme climate events threatens further damage to food and feed production [11]. Many species are and will be affected by combinations of elevated atmospheric CO_2_ concentration, increased temperatures, and changing seasonal rainfall patterns [12]. Between 2013 and 2016, for example, all Caribbean islands experienced an extensive drought that pushed more than two million people into food insecurity [13], and over 50% of the crops were lost in some of these regions [14]. Additionally, drought cost the United States of America (USA) USD $250 billion in damages, one of the costliest natural disasters [15]. Aside from natural causes, agricultural practices, such as artificial fertilization, burning agricultural residues, trading, long-distance transportation, and pesticides, are responsible for significant carbon and methane emissions and environmental pollution [16]. These factors, combined, are believed to have accelerated climate change, and there is an urgent need to adopt practices to reduce the future impacts of extreme climate events [17].

Currently, abiotic stresses, such as drought, salinity, and flooding, already limit food production severely, resulting in yearly global losses of over USD $100 billion to the agricultural sector [18,19]. Coupled with the abovementioned stresses, heavy metal accumulation and nutrient deficiencies promote hostile soils for food, feed, and fuel production [18,20]. While healthy soils are pivotal to sustainable crop yield, one-third of global soils face progressive degradation [21,22]. Therefore, effective adaptation strategies are needed to mitigate the negative impacts of these soils on crop production. As such, technology-based approaches are a faster alternative to traditional techniques and management strategies [23].

Current and developing technologies that might aid in creating or enhancing stress resilience in crops include molecular-assisted plant breeding [24], genetic manipulation of traits by transgenesis or gene editing [25,26], plant-microbe engineering [27,28], de novo domestication of wild species [29], and artificial apomixis [30]. In most of these approaches, natural genetic variability and gene orthology are sources of targets for genetic manipulation to enhance crops. Natural variation in the gene, in its *cis*-regulatory elements, protein-coding sequence, transcription start and termination sites, and splice sites, can explain intra-species variability regarding stress tolerance, for example [31]. In addition, since the genome-wide functional characterization is unavailable for any single species, researchers use the orthology-function conjecture, wherein orthologous genes might perform similar functions in different species [32]. In both cases, genetic information from intra- or interspecies variation guides the strategies to engineer desirable traits. In this context, an under-utilized genetic resource resides in crop wild relatives and extremophytes that can be naturally tolerant to extreme conditions [33]. A broader knowledge of extremophytes’ genetics could provide even more information to the plant biotechnology toolbox. 

Given this background, this review will focus on recent advances in the applications of gene editing by Clustered Regularly Interspaced Short Palindromic Repeat (CRISPR)/CRISPR-associated protein (Cas) systems in crops to cope with hostile soil conditions, such as drought, flooding, salinity, accumulation of heavy metals or toxic elements, and nutrient deficiency. Since CRISPR/Cas technologies enable the targeted and accurate genetic modification of crops without the incorporation of foreign DNA, they increase the speed of crop improvement [34] and are gaining popularity instead of classic transgenesis [35]. The use of extremophytes as reservoirs of natural variants and orthologue targets for CRISPR/Cas applications, as well as future challenges and prospects of this technology, are also discussed.

## 2. A Broad Overview of CRISPR/Cas Technologies in Plants

**Figure 1:**
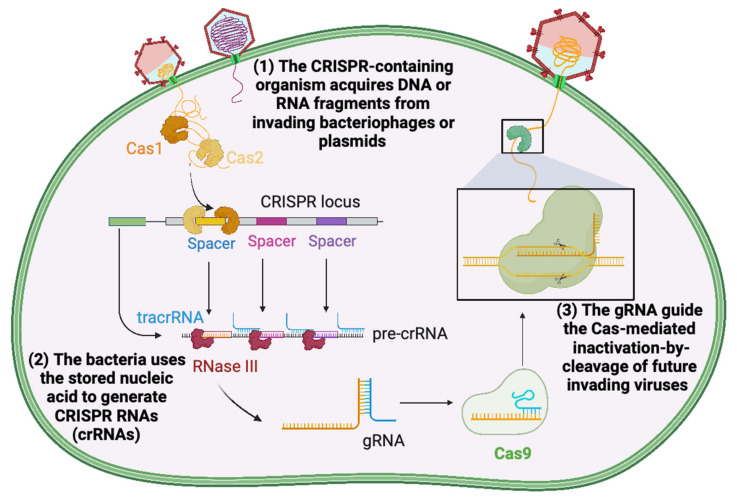
CRISPR/Cas-mediated immunity in bacteria: Three main phases. Image adapted from CRISPR-Cas9 adaptive immune system of *Streptococcus pyogenes* against bacteriophages template by BioRender.com (2023). Retrieved from https://app.biorender.com/biorender-templates, accessed on 31 March 2023.

Precise genome editing techniques and applications have radically changed after the development of CRISPR/Cas technologies. The CRISPR defense systems were first noticed in bacterial genomes in 1987 [36], as part of the natural adaptive immunity in bacteria and Archaea [37,38]. In general, CRISPR/Cas-mediated immunity occurs in three steps: (1) the CRISPR-containing organism acquires deoxyribonucleic acid (DNA) or ribonucleic acid (RNA) fragments from invading bacteriophages or plasmids, then (2) it uses the stored nucleic acid to generate CRISPR RNAs (crRNAs) to (3) guide the RNA (gRNA) toward the Cas-mediated inactivation-by-cleavage of future invading viruses (Figure 1) [39].

In 2012, Jinek et al. [37] showed that the Type II CRISPR/Cas9 system of *Streptococcus pyogenes* could be mimicked using a single chimeric gRNA instead of the natural trans-activating crRNA (tracrRNA):crRNA duplex. Similar work was concurrently published by Gasiunas et al. (2012) [40], utilizing the CRISPR/Cas9 system from the bacterium *Streptococcus thermophilus*. Both publications proposed using the artificial CRISPR/Cas9 system to induce double strand breaks (DSB) at target locations in a genome of interest and to take advantage of the error-prone DSB repair pathways to generate genetic variability. Since then, CRISPR/Cas technologies have become synonymous with a relatively cheap, global gene-editing tool, and several modifications and enhancements have been made in this first decade of use [39]. In addition, since the CRISPR/Cas system is diverse, and different combinations of Cas proteins participate in the immune process depending on the host species [41], more systems and variations can be discovered.

**Figure 2:**
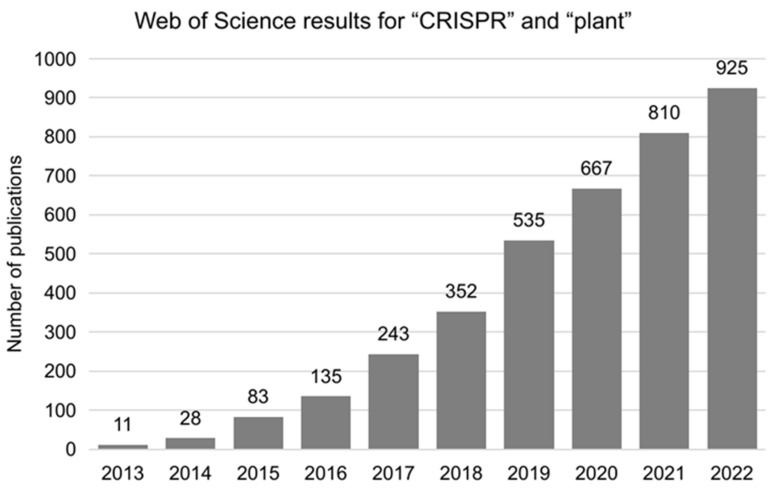
New original research papers per year in the Web of Science database (webofscience.com) from 2013 to 2021 containing the keywords “plant” and “CRISPR”.

In plants, the first five reports of CRISPR/Cas9-based genome editing were published in August 2013 and focused on demonstrating the vast versatility of the technology in the area of plant biology (proof of concept) in the model species *Arabidopsis thaliana*, *Nicotiana benthamiana*, and *Oryza sativa* [42,43,44,45,46]. Shortly after, the CRISPR/Cas9 system became a helpful tool for the functional annotation of plant genes [47], and the first reports of its use in model crops showed successful results in sorghum [48], wheat [49], maize [50], and soybean [51,52]. Since 2012, the number of publications related to genome editing in plants using this technology has grown exponentially (Figure 2), and 925 Research Articles were published in 2022 alone.

**Figure 3:**
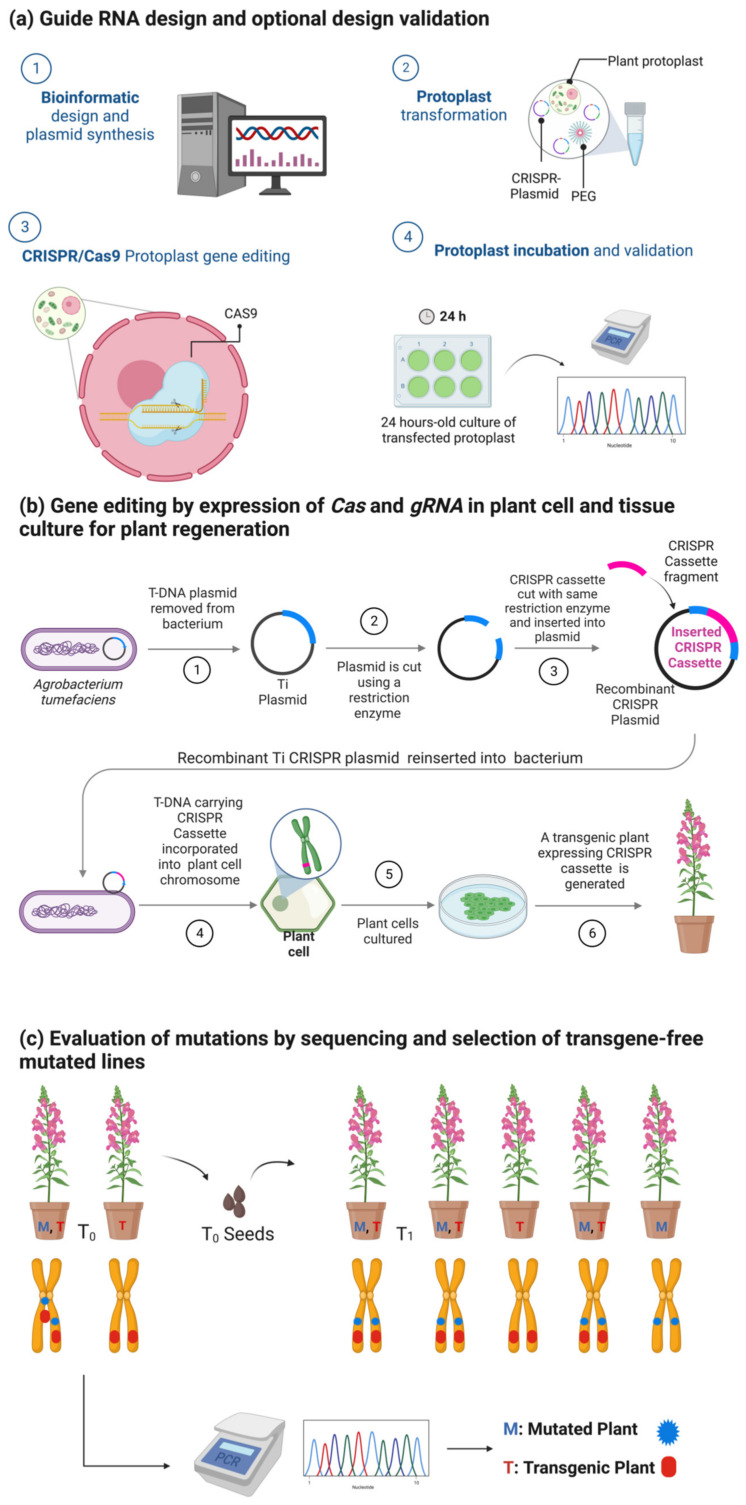
Standard protocol for generating transgene-free gene-edited plants using CRISPR/Cas9. (**a**) The use of protoplasts is the most common technique for validating CRISPR/Cas9 construct designs and generating transient gene expression. (**b**) Then, Agrobacterium-mediated transformation is the common technique to generate CRISPR/Cas9 mutated plants with a stable gene expression. (**c**) Finally, elimination of transgenic sequences is performed to generate “null segregants” via Mendelian segregation. Images (**a**,**b**) are adapted from Agrobacterium-Mediated Transformation and CRISPR-Cas9 Gene Editing in *Trypanosoma cruzi* templates by BioRender.com (2022). Retrieved from https://app.biorender.com/biorender-templates, accessed on 31 March 2023.

In the majority of these publications, the general protocol needed to achieve a CRISPR gene-edited line in plants is comprised of four main steps: (1) guide RNA design and optional design validation, (2) gene editing by expression of *Cas* and *gRNA* plant cells, (3) tissue culture for plant regeneration, and (4) evaluation of mutations by sequencing and selection of transgene-free mutated lines (Figure 3). For successful gene editing, 20 nucleotides specific to the target DNA sequence must be provided in the gRNA, applying standard RNA–DNA complementary base-pairing rules [53]. The target sites must be contiguous to a Protospacer Adjacent Motif (PAM) sequence, which varies depending on the Cas nuclease chosen [54]. In the case of the Cas9 nuclease, activity is directed to any DNA region preceding a 5′-NGG-3′ PAM sequence [55]. Several bioinformatics tools have been developed to help design gRNAs and predict their off-target potential [56]. An optional validation of the gRNA can be performed by transiently expressing the *Cas/gRNA* in plant protoplasts. In this stage, genomic DNA from the protoplast culture is submitted to sequencing to evaluate the presence of mutations in the desired region [48]. After proper gRNA design, a *Cas/gRNA* transgene is used for the genetic transformation of a target plant, using the appropriate explants and tissue culture protocols (Figure 3b). Then, the last step is to assess whether independent lines carry mutations in the target site by conventional PCR, followed by Sanger sequencing or Illumina deep sequencing. An additional step is usually added to eliminate the *Cas/gRNA* cassette to prevent off-target mutations induced by the nuclease’s constant expression and to reduce concerns about ‘genome-editing’ plants. Thus, one of the most used strategies to obtain mutant lines without the *Cas/gRNA*-expressing transgene is selection by Mendelian segregation, facilitated by visual markers, such as fluorescent proteins (Figure 3c) [57]. However, other transgene-free methods are desired, since traditional methods are laborious and time-consuming. These methods include technologies for self-elimination of transgenes, direct delivery of the *Cas/gRNA* ribonucleoprotein (RNP), or expression of *Cas/gRNA* by viral vectors [58].

Following the success of CRISPR/Cas technologies in single gene editing, several modifications were developed to enhance and diversify their use [39,59]. The multiplex CRISPR approach is a strategy that enables the editing of multiple genes in a single transformation event and uses multiple gRNAs delivered at once to achieve this goal [60,61,62,63]. Abdallah et al. [60] used three distinct gRNAs to knockout five TaSal1 genes in wheat, resulting in drought-tolerant seedlings. In another example, Lorenzo et al. [64] used 12 gRNAs, in combination, to target the knockout of 12 different growth-related genes, producing lines with enhanced yield in maize. Another approach is to produce point mutations using CRISPR base editing, which does not create breaks in the DNA. This technique uses a catalytically defective Cas enzyme, with nickase activity in a single DNA strand (nCas). Then, nCas is fused with enzymes with deaminase activity that modify the bases in the window targeted by the gRNA [54]. A different technology, Prime Editing CRISPR (CRISPR-PE), allows the insertion of sequences at the target location by providing a RNA template in the gRNA (then called pegRNA) when nCas produces a single strand break. In this technique, nCas is fused with a Reverse Transcriptase that uses the template pegRNA to insert the modification on the generated single strand [54]. Other systems, such as CRISPR-Combo [65], combine gene editing capabilities with CRISPR/Cas-based gene expression activation to boost plant genome engineering. In this case, gRNAs with different protospacer lengths determine whether the target is cleaved by the Cas9 (20 nt protospacer) or activated by the MS2-SunTag activator (15 nucleotides protospacer). The authors tested several applications of the system, and one example is the concurrent activation of *AtFT*, accelerating flowering time and inactivation of herbicide target genes *AtALS* and *AtACC2*. By selecting only early flowering plants, transgene-free herbicide-resistant mutants were easily detected [65]. The different CRISPR/Cas technologies can be used in genome-wide screens, which provide a targeted approach in generating a large number of mutants that can be selected by their phenotype in a desired condition [66]. The identification of causal genes is then easily performed by using the gRNAs as barcodes in deep sequencing [67]. Gaillochet et al. [66] and Pan et al. [68] review CRISPR screening in plants in more detail, demonstrating its potential to identify genes and generate varieties tolerant to multiple stresses, since mutants can be selected by phenotype after they are established. The toolbox of CRISPR/Cas technologies is still expanding, and this plethora of strategies to manipulate plant genomes shows promise to generate transgene-free stress-resilient crops.

From this perspective, genome manipulation techniques have always supported basic and applied crop research and are crucial for modern agricultural production [69]. Although these techniques require prior physiology and molecular genetics knowledge of the plant species under study, at least 42 plant species have been successfully edited by CRISPR/Cas technologies [70]. An attractive characteristic of CRISPR/Cas systems is their success in genome editing polyploid species, which is the case for most crop and biofuel species [71]. With the theoretical knowledge and the know-how of gene-editing techniques, custom modifications can be targeted to specific genes to improve desired traits in a highly predictive manner. The following sections summarize stress-related genetic discoveries in crops achieved by CRISPR/Cas and discuss the potential of these technologies for engineering crops with higher tolerance to extreme conditions affecting the soil–plant interface.

## 3. Advances in Engineering Commercial Crop Genomes to Cope with Different Hostile Soil Conditions

The decline in soil quality poses a significant challenge to agriculture [72], and CRISPR/Cas systems can be valuable tools to address abiotic stress-related traits in plants. These traits are often controlled by regulatory genes, which can be knocked out or down to improve tolerance [73]. However, natural environments typically present a combination of different stresses simultaneously, and while most progress has been made in studying individual stresses, genome-wide association studies and transcriptomic information have identified numerous candidate genes involved in stress tolerance regulation that are potential targets for CRISPR/Cas applications [74]. Cross-species analysis of stress responses shows a conserved core genetic response to stresses [75,76,77], which may help develop genotype-independent strategies to cope with a changing climate. CRISPR-edited single genes that confer tolerance to individual stresses can be used as a starting point for a multiplexed approach, where combinations of mutations can confer combined stress tolerance.

### 3.1. Drought Stress Tolerance

Water deficiency is a chronic abiotic crop stress that impacts plant growth and development, constituting about 70% of potential crop yield and productivity losses globally [78]. Drought exists either due to significantly less rainfall or a significant decrease in the quantity of moisture, and it is considered a substantial abiotic stress, hindering agriculture and forestry [78]. Indeed, modeled climate change projections show an even worse scenario for drought, independent of the decrease or increase in greenhouse emissions in most of the world [79,80]. Soil drought can have significant negative impacts on crops by reducing plant growth, altering plant architecture, delaying or inhibiting plant development, reducing reproductive success, and increasing susceptibility to diseases and pests [78]. 

Plant responses to drought might be classified in five critical processes: sensing, avoidance, tolerance, scape, and recovery. After drought sensing, two pathways can be activated: an abscisic acid (ABA)-dependent pathway or an ABA-independent pathway, triggering the activation of transcription factors and specific drought responsive genes [81,82]. Drought avoidance involves morpho-physiological changes, such as stomatal closure, leaf area or leaf number reduction, wax synthesis, and increased root systems [82]. On the other hand, drought tolerance involves mechanisms to cope with severe drought at different phenological stages, such as changes in stomatal density, gene expression of drought responsive genes, and synthesis of osmoprotectans. In addition, the reduction of photosynthesis rate under drought leads to an imbalance in energy, inducing the production of Reactive Oxygen Species (ROS), which are signaling molecules of stress that can damage the plant cellular machinery [83]. An additional tolerance mechanism is the biosynthesis of antioxidant molecules and expression of the enzymatic antioxidant system to ameliorate the oxidative cellular stress triggered by drought conditions [83]. Short life cycles or early flowering, on the other hand, are examples of escape mechanism to drought, and they could be interesting targets for gene editing. Finally, recovery, the capacity of the plant to survive a severe drought event, involves processes of cellular protection, repair, and stress priming to promote photosynthesis recovery, and it has been extensively studied in resurrection plants [84]. These cellular processes are all targets for genetic manipulation, and several elements have already been studied by CRISPR/Cas technologies. A comprehensive summary of research that employed CRISPR/Cas-directed mutagenesis strategies to study drought stress tolerance in crops is presented in Table 1. All of the studies have proven to either enhance or reduce performance of the mutant plants in comparison to the wild type by experiments in growth chambers, greenhouses, or in the field. Proof-of-concept studies and cross-species gene validation studies were excluded from Table 1 for brevity. 

Most studies employing CRISPR/Cas genome editing to study drought resistance so far have occurred in rice varieties (Table 1). An in-frame deletion of a *DROUGHT AND SALT TOLERANCE* (*DST*) gene using CRISPR/Cas9 in *O. sativa* subsp. *indica* caused deletion of the C-terminal EAR motif in the protein product, which produced plants with broader leaves and reduced stomatal density in comparison with the wild type, resulting in enhanced water retention under dehydration stress [102]. The rice CONSTANS-like transcription factor, *Ghd2*, regulates drought-induced leaf senescence, and its knockout (KO) by CRISPR/Cas9 enhances drought tolerance by delaying the senescence process [98]. While these examples are of mutations that increased tolerance to drought, several CRISPR studies revealed genes whose KO impairs tolerance. The KO of gene *OsNPF8.1*, a nitrate transporter, reduced tolerance to drought and salt stress, as well as lower grain yield with less N accumulation in comparison with the control genotype [125].

Another important characteristic of CRISPR/Cas technologies is their ability to KO microRNAs, previously difficult to achieve by classic transgenesis due to their short sequence [161]. Um et al. (2022) [113] used CRISPR/Cas9 to generate KO rice lines for *osa-MIR171*, which showed sensitivity to drought in comparison to the wild type. With additional experiments, the authors show that *osa-MIR171* regulates the expression of flavonoid biosynthesis genes, which are known participants of stress response pathways [162]. Contrastingly, the CRISPR/Cas9 KO of *osa-miR535* in rice enhances the tolerance of plants to dehydration and PEG stresses in comparison to unedited plants [100]. Interestingly, *osa-mir535* is a highly conserved miRNA present in more than 50 plant species [163], which makes it an interesting target to engineer drought tolerance in crops.

In *Solanum lycopersicum* (tomato), pipecolic acid (Pip) biosynthetic gene, *SlALD1,* CRISPR-generated mutants show elevated drought resistance compared with the wild-type, a phenotype associated with CO_2_ assimilation, photosystems activities, and antioxidant enzyme activities [136]. Still, in tomatoes, the KO of the jasmonic acid-responsive transcription factor *SlLBD40* by CRISPR/Cas9 enhanced drought tolerance in comparison to unedited plants [140]. Similarly, in maize, CRISPR/Cas9 KO mutants of another LBD transcription factor, *ZmLDB5*, have higher grain yield under drought stress compared to the wild type and do not exhibit differences in well-watered conditions [151]. In soybean, a quadruple KO of circadian rhythm transcription factors *GmLHY1a*, *GmLHY1b*, *GmLHY2a*, and *GmLHY2b* produced plants with enhanced drought tolerance and delayed maturity in comparison to the unedited genotype [89]. It is important to note that, although most CRISPR/Cas research so far has focused on model crops, such as rice and maize, other species are being explored, including oilseed rape [85], cucumber [86], strawberry [87], alfalfa [94], tobacco [95,96,97], poplar [133,134,135], potato [144], wheat [145,146,147,148], and grape [149] (Table 1). Collectively, these studies provide a growing database of mutant alleles that modulate responses to drought in crops, which could be used to breed stress-resilient cultivars. Furthermore, based on orthology principles, similar mutations could be effective across different species.

### 3.2. Flooding and Waterlogging Tolerance

Although drought and flood are viewed as opposing stresses and are usually studied separately, they share molecular pathways of tolerance, and both stresses reduce energy-consuming processes, facilitating the allocation of energetic resources to stress adaptation [164,165]. Similar to plants in drought stress, shoots need to adapt to dehydration caused by impaired root hydraulics and leaf water loss after a flood event [165]. Due to climate change, there is evidence suggesting that compound extremes, such as the co-occurrence of droughts and floods, are increasing in many parts of the world, including the United States, Europe, and Asia [166]. When the El-Niño South Oscillation perseveres, for example, it leads to prolonged flooding in some areas [80], and drought–flood abrupt alternation is becoming more unpredictable [167]. The co-occurrence of droughts and floods can amplify their impacts and create complex challenges for ecosystems, agriculture, water resources, and human settlements.

In addition to flooding, characterized by the presence of standing water above the soil surface, another phenomenon of excess water in the soil is waterlogging, which is the lack of drainage [168]. Both flooding and waterlogging can cause significant damage to crops and soil, but they have different impacts on plant growth and development [169]. While some plants may be adapted to tolerate occasional flooding, most plants are highly sensitive to waterlogging and can suffer from reduced growth, root damage, and even death [170]. Unfortunately, until the publication of this review, no studies involving CRISPR/Cas have tackled waterlogging, and only two examples of specific flood-related CRISPR studies were found (Table 2). In both cases, the evaluated gene KOs present reduced tolerance to floods. The *OsGF14h* gene encodes a 14-3-3 protein in weedy *O. sativa* subsp. *japonica* cultivar WR04-6, and its KO mutant in this background is sensitive to anaerobic conditions imposed by flooding stress. Interestingly, already sensitive modern cultivars SN9816 and Nipponbare show six polymorphic sites in the coding sequence of *OsGF14h*, which produce an incomplete isoform of the 14-3-3 protein [171]. The second study knocked out the ethylene-response factor-like gene *SUB1A* in a flooding-tolerant cultivar of *O. sativa* subsp. *indica*, Chiherang-Sub1, resulting in sensitivity to the flooding experiment, similar to the wild-type cultivar Chiherang [172]. Another interesting study by Ye et al. [173] verified that CRISPR-mediated KO of gene *OsCBL10* is embryo-lethal, but natural variations in the gene’s promoter were associated with flooding tolerance.

In summary, since drought and flood may coexist and possibly share regulatory mechanisms in plants, it is urgent to revisit already characterized mutants tolerant to drought concerning flood tolerance. This would be an effective strategy to accelerate the discovery of genes conferring this trait. Furthermore, genetic resources for flood tolerance might be found in crop wild relatives. All major crop families possess members that show adaptation to seasonal wetlands, including members of genera *Oryza* and *Zea* (*Poaceae*), *Lotus* (*Fabaceae*), *Solanum* (*Solanaceae*), and *Rorippa* (*Brassicaceae*), which can provide insight into plastic survival strategies lost during crop domestication or selection for production agriculture [174].

### 3.3. Salinity Stress Tolerance

Another global problem in agriculture, affecting over 400 million ha worldwide, with direct implications on crop yield and food security, is soil salinity [72]. This phenomenon can be caused by irrigation with saline water or over-irrigation [175], excessive fertilization [176], conversion of natural habitats to agricultural land [177,178], geological factors [179], climate effects, sea level increase, flooding, or tsunamis [180,181]. The effects of salinity on crops have been extensively studied at the physiological and molecular levels, including osmotic and toxic consequences on different phenological stages [182,183,184]. 

Physiological consequences of salinity stress include ion toxicity, which impairs the uptake and transport of essential nutrients [185], osmotic stress, which reduces water potential in cells [186], oxidative stress [187], changes in the expression of genes involved in growth and development [188], and alteration of hormones impacting growth and stress responses [189]. At the early phase of perception, sodium/hydrogen exchangers (NHXs) and high-affinity potassium transporters (HKTs) import Na^+^ ions and activate a Na^+^ sensing module [190]. Then, early signaling is activated, involving K^+^, Ca^2+^, cGMP, phospholipids, ROS, and protein kinases that can activate hormones and gene responses downstream [191].

This signal cascade allows the expression of different adaptive mechanisms, such as growth and developmental response, ion exclusion and sequestration, and the synthesis of compatible solutes to cope with osmotic stress [183]. Some tolerant plant species have developed specific mechanisms to excrete salt ions through specialized structures [192]. Other species can induce the synthesis of osmoprotectant metabolites, such as proline, glycine betaine, γ-GABA, spermidine, spermine, putrescine, mannitol, sucrose, trehalose, and enzymatic and non-enzymatic antioxidant molecules [193,194,195,196].

**Figure 4:**
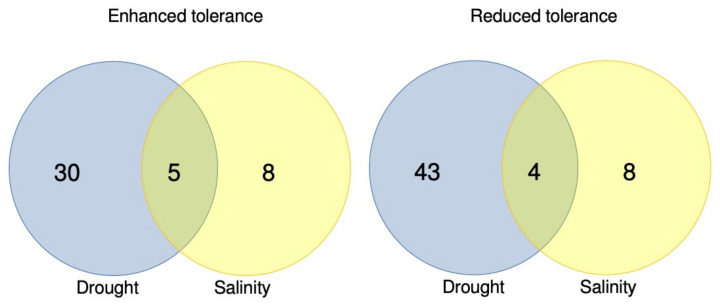
Overlap between genes involved in drought and salinity stresses in CRISPR studies presented in this review. There are a total of 35 studies of gene editing promoting enhanced drought tolerance, of which five overlap with enhanced salinity tolerance. Studies of reduced drought tolerance sum to 47, of which four also show reduced salinity tolerance. For salinity, a total of 13 genes shows enhanced tolerance, while 12 show reduced tolerance.

Salinity stress is the second most common abiotic stress with available CRISPR/Cas data, with many of the same genes also implicated in drought tolerance (Table 3, Figure 4). There are five genes with CRISPR mutants that enhance both drought and salinity tolerance: *OsPPR035* and *OsPPR406* [109], *OsDST* [102], *osa-MIR535* [100], and *OsIPK1* [105], all in rice. There are also four genes with mutants having reduced stress tolerance: *OsNPF8.1* [125] and *OsDIP1* [119], in rice, as well as *GmMYB118* [91] and *GmCOL1a* [90], in soybean. This overlap is largely explained by the shared genetic networks involved in the ABA-dependent and ABA-independent pathways of the abiotic stress response [197]. The CRISPR/Cas9-generated in-frame deletion of 33 bp in gene *OsIPK1* controlled the synthesis of phytic acid and conferred salt and drought tolerance without apparent penalties in yield [105]. The expression of ABA-independent TF *OsDREB1A* is upregulated in both stresses in *osipk1_1* mutants, corroborating the overlap between shared stress regulation.

In addition to the shared genes, 16 other studies are summarized in Table 3. One of these studies in rice generated 14 CRISPR-mediated mutations in gene *OsRR22*, a B-type response regulator TF involved in cytokinin signal transduction and metabolism [198]. These mutations confer salt tolerance at the seedling and mature stages compared with wild-type plants, without effects on other agronomic traits [199]. In soybean, CRISPR/Cas9 was used to validate the participation of TF *GmNAC06* in salt stress, since KO mutants display poor performance under experimental conditions in comparison to the wild-type and overexpression lines [200]. Contrastingly, enhanced performance in laboratory and field salinity stress experiments was found for double and quadruple KO soybean mutants *gmaitr36* and *gmaitr23456*, respectively [201]. This was achieved by a multiplexed approach of CRISPR/Cas9-mediated KO of *GmAITR* genes, which are ABA-induced transcription repressors involved in regulating ABA signaling [202].

**Table 3:** Studies employing CRISPR/Cas on genes related to salinity stress. TF: Transcription Factor; ABA: Abscisic Acid; KO = Knockout; KD = Knockdown; I.N.F. = Information Not Found.

Species	Target Locus	Pathway/Function	Effect on Tolerance	Result	Reference
*Cucurbita moschata*	*CmoPIP1-4*	Plasma membrane intrinsic protein	Reduced	KO	[203]
*Glycine max*	*GmAITR2* *GmAITR3* *GmAITR4* *GmAITR5* *GmAITR6*	ABA-induced transcription repressor	Enhanced	KO	[201]
	*E2*	Photoperiodic flowering	Enhanced	KO	[204]
	*GmCOL1a*	CONSTANS-like TF	Reduced	KO	[90]
	*GmMYB118*	MYB TF	Reduced	Amino acid change	[91]
	*GmNAC06*	NAC TF	Reduced	I.N.F.	[200]
*Hordeum vulgare*	*HVP10*	Vacuolar H+-pyrophosphatase	Reduced	KO	[205]
*Oryza sativa*	*osa-MIR535*	Drought-induced miRNA	Enhanced	KO	[100]
	*OsbHLH024*	bHLH TF	Enhanced	KO	[206]
	*OsDST*	Zn Finger TF	Enhanced	Domain deletion	[102]
	*OsIPK1*	Inositol 1,3,4,5,6-pentakisphosphate 2-kinase	Enhanced	11-amino acid deletion	[105]
	*OsPPR035*	Chloroplast RNA editing	Enhanced	KO	[109]
	*OsPPR406*	Chloroplast RNA editing	Enhanced	KO	[109]
	*OsRR22*	B-type RR TF	Enhanced	KO	[199]
	*OsVDE*	Xanthophyll cycle/Violaxanthin deoxidase	Enhanced	KD	[207]
	*BEAR1*	bHLH TF	Reduced	KD	[208]
	*OsDIP1*	TF-interacting protein	Reduced	KO	[119]
	*OsGLYI3*	glyoxalase	Reduced	KO	[209]
	*OsNPF8.1*	Peptide transporter	Reduced	KO	[125]
	*OsWRKY28*	WRKY TF	Reduced	KO	[210]
	*OsWRKY54*	WRKY TF	Reduced	KO	[211]
*Solanum lycopersicum*	*AIT1.1*	ABA transporter	Enhanced	KO	[212]
	*SlABIG1*	HD-ZIP II TF	Enhanced	KO	[213]
	*SlHyPRP1*	Hybrid Proline-rich protein	Enhanced	Domain deletion	[214]
	*Put2*	Polyamine uptake transporter	Reduced	KO	[215]

### 3.4. Heavy Metals or Toxic Element Tolerance

Heavy metals occur naturally in the Earth’s crust, and the release of these metals into the soil can occur due to natural or anthropogenic processes. Some of the natural causes of heavy metals in soils are the weathering of parent rock, releasing trace amounts of metals, transport of heavy metals from one location to another during floods, landslides or wind erosion, and atmospheric deposition from volcanic emissions, among others [216]. The biological activity of microorganisms, plants, and animals can also concentrate heavy metals in the soil through biological processes, such as uptake and bioaccumulation [217]. Anthropogenic heavy metal accumulation in soils is far more significant than natural sources, and the use of agrochemicals is the most impactful [217]. Fertilizers, pesticides, and herbicides can contain these harmful molecules, as well as cause soil acidity and erosion, which intensifies their accumulation in soils and possibly contaminates the water table [218,219]. 

The accumulation of heavy metals and toxic elements in plant tissues can affect their nutritional quality, making them unsuitable for consumption or even harmful to human health. This is a significant concern in the food industry and public health because of the reported diseases associated with the consumption of heavy metal-contaminated foods and exposure to contaminated environments [220,221]. In mining countries, such as Chile, the accumulation of heavy metals derived from the copper industry, for example, has generated the contamination of soils and groundwater in localities considered nowadays as "sacrifice zones", such as Puchuncaví and Quintero-Ventanas Bay [222]. Besides, other mining-associated activities or agricultural practices, such as smelting, industrial exhaust, irrigation with mining wastewater, natural presence in some agricultural soils, and applying fertilizers and pesticides with heavy metal traces, have generated a similar problem worldwide [223]. The primary heavy metals and metalloids found in contaminated soils are copper (Cu), zinc (Zn), lead (Pb), cadmium (Cd), mercury (Hg), and arsenic (As). In southern China, for instance, the analysis of rice samples from contaminated or very industrialized areas showed a high percentage (56 to 87%) of samples contaminated with Cd [224]. Additionally, since rice is the second-most produced staple food worldwide, its contamination generates concern and health risks in different countries [223]. 

Since heavy metals and toxic elements can have negative impacts on crop growth and development, they can accumulate in plant tissues and lead to reduced yield, quality, and even plant death [225]. These contaminants can also affect nutrient uptake and interfere with photosynthesis, respiration, and transpiration [226]. Physiological and molecular impacts on plants may lead to growth inhibition, chlorosis, necrosis, reduced photosynthesis, and decreased crop yield. These elements can also affect the uptake and transport of essential nutrients, leading to nutrient imbalances and deficiencies. At the molecular level, heavy metals and toxic elements can induce oxidative stress, disrupt cellular homeostasis, alter gene expression, and impair enzymatic activities [227]. Additionally, heavy metals and toxic elements can alter the composition and diversity of the plant-associated microbial communities, affecting plant–microbe interactions and nutrient cycling in the soil [228]. 

Some metal elements are essential micronutrients for the enzymatic cellular machinery to function. However, under an unbalance of heavy metal homeostasis, some plant species have developed mechanisms to deal with the rise of their concentrations in different cellular compartments [229]. Among the mechanisms involved, we can mention the expression of Heavy Metal ATPases (HMA) proteins [230], Zn and Fe-regulated Membrane Transporter (ZIP) proteins [231], Cation Diffusion Facilitator (CDF) proteins [232], Cation/hydrogen Exchangers (CAX) proteins [233], High-affinity Copper Transport (COPT) proteins [234], Natural Resistant Associated Macrophage (NRAMPS) proteins [235], the bHLH TFs [236], and low molecular weight chelators and subcellular sequesters, such as metallothioneins, phytochelatins, amino acids, nicotinamides, glutathione, and defensins [237,238].

Most of the genes encoding the expression of the aforementioned proteins are potential targets for CRISPR/Cas9 modification to modulate heavy metal tolerance. Although major efforts have been performed using the advances in omics tools, to identify molecular targets controlling heavy metal tolerance in plants [239], few studies show heavy metal tolerance modification for major crops (Table 4). The modulation of a plant’s response to this stress depends on its application. For phytoremediation, the goal is to increase the uptake of heavy metals from highly contaminated lands, while avoiding accumulation in final food products requires a decrease in the uptake of these molecules. In rice, for instance, the KO of Cd/Mn transporter *OsNRAMP5* confers Cd tolerance to a wide range of external Cd concentrations, producing shoots with sufficient nutrients and grains with lower Cd accumulation [240]. The KO of *OsNRAMP5* in two *O. sativa* subsp. *japonica* varieties generated lines with decreased accumulation of Cd in aerial organs, but reduced yield in comparison to unedited plants in both hydroponic and field experiments [241]. Similarly, KO of the rice Low Cadmium (*OsLCD*) gene also diminished Cd accumulation in the shoot, but maintained yield under high Cd concentrations in comparison to the wild genotype [242]. Another Cd-related gene, *Sl1*, was knocked out in tomatoes, and edited plants displayed increased Cd accumulation in plant tissues, as well as increased ROS activity in comparison to the wild-type and overexpression lines [243].

The *R2R3 MYB* transcription factor *OsARM1* regulates arsenic(As)-associated transporter genes, and KO lines generated by CRISPR/Cas9 improve the tolerance of rice to As in comparison to the wild-type [244]. A similar proof of concept used the KO of Antioxidant Protein 1 (*OsATX1*) gene, a Cu chaperone in rice, which induced an increase in Cu concentration in roots, thereby decreasing the root-to-shoot translocation of Cu [245]. The CRISPR/Cas technology has also been used to deal with other toxic element contamination in soils, such as radioactive Cs+. The inactivation of Cs+ transporter *OsHAK1* in rice by CRISPR/Cas9 dramatically reduced the uptake of Cs+ in highly Cs+ contaminated lands from Fukushima, Japan [246].

**Table 4:** Studies employing CRISPR/Cas on genes related to flooding heavy metal and toxic element stresses. TF: Transcription Factor; KO = Knockout.

Species	Target Locus	Pathway/Function	Effect on Tolerance	CRISPR Result	Reference
*Oryza sativa*	*OsHAK1*	Cs+-permeable transporter	Cesium resistant	KO	[246]
	*OsATX1*	Cu chaperone	Dosage-dependent tolerant	KO	[245]
	*osa-MIR535*	Drought-induced miRNAs	Enhanced	KO	[247]
	*OsARM1*	R2R3 MYB TF regulator of As-associated transporters genes	Enhanced	KO	[244]
	*OsLCD*	Unknown, Cd related	Enhanced	KO	[242]
	*OsLCT1*	Low affinity cation transporter	Enhanced	KO	[248]
	*OsNRAMP1*	Cd and Mn transporter	Enhanced	KO	[249]
	*OsNRAMP5*	Cd and Mn transporter	Enhanced	KO	[240,248]
	*OsPMEI12*	Pectin Methylesterase	Enhanced	KO	[250]
*Solanum lycopersicum*	*Sl1*	E3 Ubiquitin ligase	Reduced	KO	[243]

### 3.5. Tolerance to Barrenness

Nutrient deficiencies in soils can be triggered by a variety of factors, such as soil pH and soil organic matter, which influence the types and the abundance of essential nutrients, respectively [251]. Other factors, such as soil texture, can affect nutrient availability. Soil compaction can affect the nutrient and water access by the root system, and excessive plant uptake causes nutrient depletion in soils that are heavily cropped or in which fertilization is inadequate [252]. Agricultural practices that can lead to soil barrenness or degradation include the overuse of chemical fertilizers, leading to nutrient imbalances and soil acidification [253], as well as monocultures, which can deplete soil nutrients, leading to reduced yields and increased susceptibility to pests and diseases [254]. Moreover, soil erosion results in a loss of soil organic matter, nutrients, and soil structure, leading to reduced productivity and increased vulnerability to drought and flooding [255]. In addition, pesticide use can harm beneficial microorganisms and disrupt soil food webs, leading to reduced soil fertility and productivity over time [256]. Additionally, the use of fertilizers to boost the yield of crops has allowed for maintaining the requirements for global food security during the years past the green revolution. However, this practice is under the threat of actual climate change and geopolitical sceneries [257,258]. However, the environmental pollution and ecological degradation generated by the indiscriminate use of fertilizers [259], as well as the fertilizers’ price increment generated by recent events, such as the Russian-Ukraine conflict [258], will raise the cost of the farmer’s production, making this practice unsustainable over time, as we know today. Finally, natural disasters, such as floods, droughts, and wildfires, can also contribute to soil barrenness by altering soil properties and reducing nutrient availability [260].

Nutrient use efficiency (NUE) is the capability of a crop to take up the nutrients from soil, transport them, assimilate them, and use them to maximize its yield. NUE is a very complex trait, involving several plant functions and metabolic pathways. The polyploidy nature of major crops makes their manipulation a big challenge for plant researchers. Nevertheless, some studies have been performed to improve NUE using transgenic [261], siRNA [262], and gene over-expression approaches [263,264], which have shown impressive advances focused on NUE. Nowadays, physiological and genomic information can be used to select targets for CRISPR/Cas NUE improvement, showing promising results. Recent thorough reviews were published on potential targets for nutrient use efficiency [265,266,267], and more CRISPR-based studies might benefit from this knowledge. In total, eight studies are summarized in Table 5, including one in barley, five in rice, one in *Populus*, and one in wheat.

For example, a CRISPR cytosine base editing system (CBE) was used to generate a C-T point mutation in gene *OsNRT1.1B* of Japonica rice cv. Nipponbare, causing amino acid conversion T327M [268]. This mutation corresponds to an allele difference between rice varieties Nipponbare (T327) and IR24 (M327) [269], and the base editing conversion of the Nipponbare allele results in better NUE in comparison to the wild type [268,269]. Interestingly, the mutated DST protein in the Indica rice cv. MT1010 *dst* mutant shows enhanced drought and salinity tolerance [102], while its CRISPR KO in Japonica rice cv. ZH11 impairs NUE in comparison with the wild type, showing reduced growth in nitrogen-poor substrates [108].

In another major staple food crop, wheat, lines with mutant alleles of Abnormal Cytokinin Response 1 Repressor 1 Protein (TaARE1) were generated by CRISPR/Cas9, showing increased NUE, delayed senescence, and higher grain yield than the wild-type [270]. The same orthologous gene in barley, *HvARE1*, was mutated by CRISPR/Cas9, generating improved NUE in mutant lines *1are1-E-7-6* (amino acid substitution E78G) and *2are1-K-4* (substitution N205D) [271]. Recently, the overexpression of the *PdGNC* transcription factor in poplar was found to increase nitrate uptake, remobilization, and assimilation, improving overall NUE in this species, which was validated using CRISPR/Cas9 mutants [272].

**Table 5:** Studies employing CRISPR/Cas on genes related to nutrient deficiency stress. TF: Transcription Factor; KO = Knockout.

Species	Target Locus	Pathway/Function	Effect on Tolerance	Result	Reference
*Hordeum vulgare*	*HvARE1*	Abnormal cytokinin response 1 repressor 1 protein	Enhanced	Amino acid change	[271]
*Oryza sativa*	*NRT1.1B*	Nitrogen transporter gene	Enhanced	Base editing	[268]
	*OsDST*	Zinc finger TF	Reduced	Domain deletion	[108]
	*OsNPF3.1*	Nitrate/Peptide transporter	Reduced	KO	[273]
	*OsNPF8.1*	Nitrate/Peptide transporter	Reduced	KO	[125]
	*OsNR1.2*	Nitrate/Peptide transporter	Reduced	KO	[108]
*Populus* clone 717-1B4 (*Populus tremula* × *Populus alba*)	*PdGNC*	Nitrate uptake	Reduced	KO	[133]
*Triticum aestivum*	*TaARE1-A* *TaARE1-B* *TaARE1-D*	Abnormal cytokinin response 1 repressor 1 protein	Enhanced	KO	[270]

## 4. Extremophytes: Genetic Reservoirs for CRISPR/Cas Applications

Although evolutionarily distant species may exhibit different transcriptional responses to stress, they share core genetic regulatory elements [75]. In this context, studying extremophytes’ genetics poses a great opportunity to find potential targets for genetic manipulation, leading to enhanced stress-related traits. Extremophiles can be defined as organisms capable of dealing with extreme conditions of pH, temperature, pressure, salinity, high concentrations of gasses (such as CO_2_), metals, and ionizing radiation, for example [274,275]. The first well-studied extremophiles are microorganisms, which have already been extensively used in the bioprospection of potentially valuable enzymes, mainly in the biofuel industry [276]. A classic example is the DNA polymerase isolated from the thermophilic bacterium *Thermus aquaticus* [277], an essential enzyme in molecular biology research. Another example is the use of extremophile microbiota that induce drought/salinity resistance in plants, which have been isolated from deserts [278] and Antarctica [279]. Although there is high interest in extremozymes, bioactive compounds, and cultured extremophiles for direct use in the industry [280], little has been explored in plant genetic engineering.

Extremophyte species grow in harsh conditions, which are limiting to unadapted species. For instance, propagules of sub-Antarctic species may arrive in more extreme Antarctic regions, but few can establish new individuals that survive more than one season, and none can establish populations without human intervention [281]. This unique feature of extremophytes defies the trade-off between growth and stress resilience, since they can properly balance their resources, obtained from photosynthesis, to adapt to the extreme climatic factors to complete their life cycles [282]. In this context, deserts (warm and cold), salt pans, geothermal springs, and high mountains, common niches of extremophytes, serve as excellent model conditions to study plant performance on hostile soils [283]. 

Unfortunately, studies on the molecular and physiological determinants of the trade-off between growth and stress tolerance are scarce, particularly in non-model species. This gap leaves a significant source of variation for photosynthetic functioning and stress tolerance unexplored. Therefore, the unique opportunity provided by extremophytes to investigate how they differentially invest their photosynthetic resources to adapt their life cycles under extreme climatic factors can be leveraged to understand the mechanistic bases of the trade-off between productivity and stress tolerance [282,284]. Moreover, extremophytes offer a promising source of valuable traits for the biotechnology industry to improve crop productivity, as well as at least to maintain it in agricultural regions affected by climate change scenarios [280,285]. As the climate changes, extremophytes can provide insights into the future. Discovering the molecular and biochemical adaptations employed by these plants can enhance our understanding of how plants, in general, will respond to climate change [286].

Interestingly, even though molecular mechanisms controlling plant physiology during abiotic stress have been amply reviewed in model plants and crops [287,288], our knowledge of the molecular mechanisms that support extremophytes success is more limited [275]. Some of the best-studied extremophytes are the resurrection plants, for their potential as ideal models to engineer crops with enhanced drought tolerance [289,290]. Similarly, many studies have been performed on halophyte plants, including highly salt-tolerant close relatives of *A. thaliana*, allowing for direct comparisons of stress tolerance mechanisms [3]. Since established protocols for greenhouse cultivation, in vitro culture, and transformation or gene editing of extremophytes are scarce, functional genetic studies have mostly focused on the heterologous expression of extremophile proteins in model plants [31]. For instance, HIGH-AFFINITY POTASSIUM TRANSPORTER (*HKT*) genes from the halophytes *Thellungiella salsuginea* [291], *Eutrema parvula* [292], and *Suaeda salsa* [293] have been expressed ectopically in *Arabidopsis* plants, and they confer salt tolerance in comparison to the wild-type protein.

**Figure 5:**
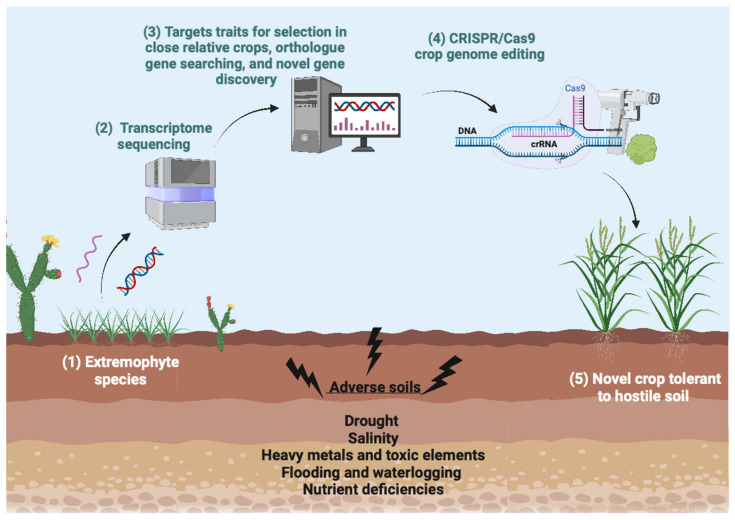
Schematic depicting the use of extremophytes and new sequencing technologies to identify new gene targets that can be modified in crops using CRISPR/Cas9. Image created with BioRender.com, accessed on 31 March 2023.

Transcriptome sequencing is another strategy to study the reprogrammed metabolism observed in some extremophytes, enabling target trait selection in close relative crops. All major crop families possess members that show adaptation to hostile soils, including members of genera *Oryza* and *Zea* (*Poaceae*), *Lotus* (*Fabaceae*), *Solanum* (*Solanaceae*), and *Arabidopsis*, *Rorippa* (*Brassicaceae*) [174,294,295]. These species can provide insights into plastic survival strategies to hostile conditions, which were lost during crop domestication or selection for intensive agriculture. The identification of the genetic factors controlling stress tolerance traits in extremophytes can guide the search for orthologs in closely related crops, which would then be modified by CRISPR/Cas technologies (Figure 5). 

For instance, the transcriptomic analysis of *Populus euphratica*, a desert tree related to the commercial species poplar, showed a reprogrammed metabolism under salt stress, where genes involved in ABA regulation are differentially expressed [296]. Thereby, negative regulators of stress tolerance previously identified in extremophytes could be knocked down using a CRISPR/Cas system. Meanwhile, the sequences of promoters or positively regulatory regions of stress response genes could be modified, as was shown for the generation of HDR-based editing to produce a salt-tolerant SlHKT1;2 alleles in tomato utilizing the CRISPR/Cpf1-geminiviral replicon technique [297]. In another example, CRISPR/Cas9 KO of metallophyte *Sedum pumbizincicola* Heavy Metal ATPase 1 (*SpHMA1*) helped to characterize the function of *SpHMA1* in protecting PSII from Cd toxicity [298]. Therefore, there is still much to be explored and discovered in these extremophile species that can be used for crops to face the challenges of climate change and hostile soils.

## 5. Challenges and Prospects

### 5.1. Combined Stresses

Although great advances have been made in the study of stresses, most of these discoveries assessed plant responses to single stresses. In natural conditions, the combination of stresses is usually the norm, and climate change will also affect the intensity and frequency of these compound stresses [166]. Given these statements, it is possible to assume that combined stresses complicate the equation for stress resilience engineering. However, elegant systems, such as BREEDIT [64], aim to solve this problem by editing a combination of genes with multiplex CRISPR, resulting in additive roles in stress or yield traits. In their proof-of-concept study, a knock-out of 48 different genes involved in plant growth was conducted, combining 12 genes simultaneously, and generating over 1000 different edited lines with potential enhancements in yield [64]. If a similar strategy is used to knock out multiple genes associated with the suppression of stress tolerance, the combinations of such mutations could help establish a multi-tolerant plant line. Furthermore, sophisticated systems, such as CRISPR-Combo [65], couple gene editing with gene activation, allowing for fine-tuned metabolic engineering.

### 5.2. Technological Limitations and Potential Solutions

CRISPR/Cas systems have been widely acknowledged for their potential to improve crops through gene insertion, removal, point mutation, and gene replacement. However, their use in agricultural research is still in the early stages, with most reports constrained to proof-of-concept findings [299]. Even though CRISPR has been successfully applied in at least 42 plant species [70], there is still a need for a global mechanism that is genotype-independent. Several efforts are underway to improve the limitations of CRISPR/Cas technologies, such as limited PAM sites, off-target mutations, low HDR efficacy, and time consumption due to the *Agrobacterium*-mediated transformation system [300]. For instance, several mutated Cas enzymes opened the possibility of more diverse PAM sites with lower off-target potential [39], and slightly more efficient HDR could be achieved with CRISPR/Cas12a [301].

Regarding transformation limitations, advances have been achieved in both *Agrobacterium*-mediated and other methods, such as the use of viral vectors. The latter, although efficient, is limited by the size of the Cas-encoding sequences, which are very large [302]. Recently discovered Cas12f1 is considerably smaller, allowing for the use of viral vectors to produce gene editing without transgene integration [54]. These strategies hold promise for expanding the application of CRISPR/Cas9 in agriculture and addressing some of its current limitations. An important step forward in monocotyledonous and recalcitrant plant transformation, mediated by *Agrobacterium*, is the use of morphogenetic factors to induce somatic cells into initiating embryogenesis, thus partly circumventing the need for strenuous callus induction and regeneration studies [303,304]. Another important discovery is the newly described “cut-dip-budding” (CDB) system, which enables gene editing in previously recalcitrant species, which is the case for many crops and wild relatives [305]. The CBD system relies on the ability of plants to generate basal shoots from adventitious buds in roots, and it was already applied successfully in species where transformation was either difficult or impossible. An advantage of the CBD system is the absence of in vitro or sterile culture, since all steps can be performed directly in soil [305]. These technologies are promising for the application of CRISPR in wild relatives or extremophyte species to study gene function and to apply these discoveries in crop plants.

### 5.3. Field Evaluation of CRISPR-Modified Crops

The usefulness of the CRISPR/Cas editing techniques must be demonstrated before the large-scale distribution of any new variety possessing them [306]. However, as shown in Table S1, most studies on hostile soil tolerance in plants modified by CRISPR/Cas systems were only evaluated in the laboratory or greenhouse. Therefore, verifying whether results can be translated to crop plants grown in the field is crucial [307]. In addition, field trials provide a tremendous amount of otherwise unknown information on how plants respond to environmental changes under agricultural systems [308]. Unfortunately, the diverse landscape of legislation regarding gene-edited plants has hindered large-scale field trials, and most such tests have occurred only in China [309]. In 2018, the first field trial of a CRISPR/Cas9 gene-edited crop, *Camelina sativa*, began in Europe at the Rothamsted Research in the UK and provided a wealth of essential data and enabled the evaluation of the potential of a new trait [310]. During the experiment, the UK Department for Environment, Food & Rural Affairs reclassified gene-edited crops as GMOs, and the next field trial only occurred in 2021 [307]. Later, in 2021, field tests of low-asparagine gene-edited wheat were performed in this same research field and were essential to confirm the results observed in the laboratory [311]. Additionally, in 2021, Lee and Hutton (2021) [306] conducted field trials during three consecutive seasons using CRISPR-driven jointless pedicel, as well as fresh-market tomatoes, without detecting significant differences in fruit size yield between CRISPR-modified tomatoes and WT tomatoes [306]. Despite these studies, further field trials conducted across a broader range of regions are imperative to authenticate the scientific effectiveness of gene-edited plants and instill greater assurance and security for both producers and end consumers.

### 5.4. Regulation and Customer Acceptance

Important limitations on CRISPR/Cas-modified crops are the legal regulation of plant genome editing and consumer acceptance. Although CRISPR crops are being developed and grown globally, this trend is accompanied by legal, ethical, and policy debates. The technical limitations of CRISPR and whether existing GMO regulations should apply to CRISPR-edited crops are key issues [312]. In the scientific community, there is a belief that mutations generated by CRISPR/Cas9 are no different from those induced by nature or conventional breeding. Thus, plants created through this technology should not undergo the same regulatory processes as conventional GMOs. However, on a global scale, opinions differ, and some countries believe that CRISPR-generated crops should undergo the same regulations as GMOs before entering the market [313,314].

For instance, the United States and the European Union have different approaches to CRISPR-edited crops. The former is more permissive because they do not have to undergo the same regulatory process as GMOs, while in the European Union, they are considered GMOs. However, several countries have already regulated that plants generated through CRISPR with only InDels or homologous inserts can be excluded from GMO regulation [312,314,315,316]. Hence, the international community is considering whether certain CRISPR-edited crops can be excluded from regulatory oversight and what safety data would be required for CRISPR-edited crops to be regulated in specific countries. 

The success and adoption of gene-edited foods depend ultimately on consumer acceptance, which has been a problem for GMO foods due to misinformation. Consumers worldwide display limited understanding, misconceptions, and unfamiliarity with GMO food products [317]. Consumer acceptance of gene-edited foods varies across countries. In China, 45% of respondents (n = 835) agreed that gene-edited plant products should be allowed, compared to 36% for transgenic plant products [318]. In Brazil, producers (n = 37) are prone to planting transgenic beans (84%), and consumers (n = 100) are willing to include them in their diets (79%) [319]. In the UK (n = 490) and Switzerland (n = 505), participants expressed higher acceptance levels for genome editing than for transgenic modification. Acceptance depends on perceived benefits, scientific uncertainty, and location [320]. Acceptance levels for these technologies depend mainly on whether the application is believed to be beneficial, how scientific uncertainty is perceived, and where they reside [35,316]. Surveys, such as these, and the amount of safety data required, will affect the overall cost of regulation, an essential factor to consider when bringing new CRISPR plants to market [314,321].

## 6. Concluding Remarks

The worldwide deterioration of soil quality has emerged as a critical challenge for agriculture, compounded by the escalating impact of climate change. This looming crisis poses a significant risk to food security, particularly as we approach the year 2050. Unfortunately, there is no single solution to address the issue of hostile soils or to ensure food production in the future. Instead, an integrated, multidisciplinary approach is necessary, leveraging specific tools and solutions to mitigate the detrimental effects of hostile soils on agriculture. By combining these solutions from diverse approaches, we can potentially safeguard agriculture and ensure global food security. These tools include CRISPR/Cas technologies, which enable the precise editing of crop genomes to develop plants that are more tolerant to the stresses of hostile soils. In the past decade, this technique has demonstrated its efficacy in accurately editing the genomes of various organisms, including plants.

As reviewed here, several scientific studies have provided concrete evidence of the effectiveness of CRISPR/Cas technologies for developing crops that are tolerant to hostile soils. These studies have demonstrated successful applications of the technology in improving plant tolerance to stressors, such as drought, heavy metals, salinity, and NUE. As a result, CRISPR/Cas systems are increasingly being considered viable solutions to these agricultural challenges. Notably, a significant amount of research on using CRISPR to develop stress-tolerant crops is being conducted in China, suggesting a potential technological advantage in this area due to its legal status on gene editing organisms.

Although CRISPR technologies for genome engineering in plants are not infallible, ongoing technical advancements are addressing its limitations. Meanwhile, the regulatory landscape is becoming more lenient, allowing for greater openness towards CRISPR-mutated crops that are transgene-free and exempt from traditional GMO regulations. Additionally, consumer acceptance of CRISPR-modified products is predicted to increase, and evidence supports the continued use of this technology for plant breeders. To achieve crops tolerant to future challenges, we suggest leveraging CRISPR technology alongside advances in sequencing and the search for new genetic targets in extremophytes. These developments, alongside novel management strategies and biotechnologies, provide promising solutions for ensuring stable food security by 2050.

## Figures and Tables

**Table 1:** Studies employing CRISPR/Cas on genes related to drought stress. TF: Transcription Factor; GA: Gibberellic acid; ABA: Abscisic acid; BR: Brassinosteroid; SA: Salicylic acid; KO = Knockout; KD = Knockdown; I.N.F. = Information Not Found, KU: Knokup.

Species	Target Locus	Pathway/Function	Effect on Tolerance	Result	Reference
*Brassica napus*	*BnaA6.RGA*	Growth regulation/DELLA transcription regulator	Enhanced	Gain-of-function	[85]
	*BnaA6.RGA* *BnaC7.RGA* *BnaA9.RGA* *BnaC9.RGA*	Growth regulation/DELLA transcription regulator	Reduced	KO	[85]
*Cucumis sativus*	*CsAKT1*	Osmoregulation/K+ transporter	Reduced	KO	[86]
*Fragaria vesca*	*FvICE1*	Cold stress response/TF	Reduced	KO	[87]
*Glycine max*	*GmHdz4*	Drought stress response/HD-ZIP I TF	Enhanced	KO	[88]
	*GmLHY1a* *GmLHY1b* *GmLHY2a* *GmLHY2b*	Regulation of circadian rhythm/TF	Enhanced	KO	[89]
	*GmCOL1a*	Flowering time/CONSTANS-like TF	Reduced	KO	[90]
	*GmMYB118*	Flavonoid biosynthesis/MYB TF	Reduced	Amino acid change	[91]
	*GmNAC12*	Abiotic stress response/NAC TF	Reduced	KO	[92]
	*GmNAC8*	Nodulation, abiotic stress response/NAC TF	Reduced	KO	[93]
*Medicago sativa*	*MsSPL8*	Nodulation, growth, GA pathway/SPL TF	Enhanced	KD	[94]
*Nicotiana tabacum*	*NtAITR1* *NtAITR2* *NtAITR3* *NtAITR5* *NtAITR6*	ROS homeostasis/ABA-induced transcription repressors	Enhanced	I.N.F.	[95]
	*NtPOD63L*	Cell wall integrity/class III peroxidase	Enhanced	KO	[96]
	*NtRAV4*	Growth, development, stress response/RAV TF	Enhanced	KO	[97]
*Oryza sativa*	*Ghd2*	Grain development, flowering/CCT TF	Enhanced	KO	[98]
	*JMJ710*	Flowering time/Histone demethylase	Enhanced	KO	[99]
	*osa-MIR535*	Phosphate homeostasis, root development/Drought-induced miRNAs	Enhanced	KO	[100]
	*OsABA8ox2*	Biosynthesis of ABA/ABA hydroxylase	Enhanced	KO	[101]
	*OsDST*	ABA-dependent stress signaling/Zinc finger TF	Enhanced	Domain deletion	[102]
	*OsERA1*	BR signaling/GASA growth regulator	Enhanced	I.N.F.	[103]
	*OsFTL4*	Flowering/PEBP, florigen	Enhanced	KO	[104]
	*OsIPK1*	Growth, development, ion homeostasis/Kinase	Enhanced	11-aminoacid deletion	[105]
	*OsNAC016*	Growth, development, hormone signaling, abiotic stress response/NAC TF	Enhanced	KO	[106]
	*OsNAC092*	Biotic and abiotic stress response/NAC TF	Enhanced	KO	[107]
	*OsNR1.2*	Nitrogen metabolism/Nitrate reductase	Enhanced	KO	[108]
	*OsPPR035*	Energy metabolism, stress response/Mitochondrial RNA editing	Enhanced	KO	[109]
	*OsPPR406*	Energy metabolism, stress response/Mitochondrial RNA editing	Enhanced	KO	[109]
	*OsPYL9*	Stress responses/ABA receptor	Enhanced	KO	[110]
	*OsWRKY5*	ABA signaling/WRKY TF	Enhanced	KO	[111]
	*SRL1,2*	Root development, stress response/LRR-RLK protein	Enhanced	KD	[112]
	*osa-MIR171*	Flavonoid biosynthesis/microRNA	Reduced	KO	[113]
	*osa-MIR818b*	Stress response/Drought-induced miRNAs	Reduced	KD	[114]
	*OsADR3*	Spikelet development/MADS-box TF	Reduced	KO	[115]
	*OsASLRK*	Root development/Armadillo-like Repeat Kinesin	Reduced	KD	[116]
	*OsbZIP86*	Stress response/bZIP TF	Reduced	KO	[117]
	*OsCCR10*	Biosynthesis of lignin/cinnamoyl-CoA reductase	Reduced	KO	[118]
	*OsDIP1*	Root water uptake/Aquaporin	Reduced	KO	[119]
	*OsFTIP6*	Flowering, leaf senescence, plant architecture/Florigen transporter	Reduced	KO	[120]
	*OsGRP3*	RNA processing/Glycine-rich RNA-binding protein	Reduced	KO	[121]
	*OsHB22*	Growth, development, abiotic stress response/HD-ZIP TF	Reduced	KO	[120]
	*OsMYB60*	Osmoprotectants and antioxidants biosynthesis/MYB TF	Reduced	KO	[122]
	*OsMYBR57*	Drought stress response/MYB-Related TF	Reduced	KO	[120]
	*OsNAC006*	Abiotic stress response/NAC TF	Reduced	KO	[123]
	*OsNAC17*	Development, stress response/NAC TF	Reduced	KO	[124]
	*OsNPF8.1*	Nutrient acquisition/Phosphate transporter	Reduced	KO	[125]
	*OsPM1*	Ion homeostasis/Plasma membrane protein	Reduced	KO	[126]
	*OsPUB67*	Protein degradation, root development/U-box E3 ubiquitin ligase	Reduced	KO	[127]
	*OsRINGzf1*	Protein degradation/RING zinc finger E3 ligase	Reduced	KO	[128]
	*OsRNS4*	Biotic and abiotic stress response/S-like RNAse	Reduced	KD	[116]
	*OsSAPK2*	Stress/ABA–activated protein kinase	Reduced	KO	[129]
	*OsSAPK3*	Stress/ABA–activated protein kinase	Reduced	KO	[130]
	*IPA1/OsSPL14*	Growth, development, environmental stimuli response/SPL TF	Reduced	KO	[131]
	*OsAO3*	ABA biosynthesis/Aldehyde oxidase	Reduced	KO	[132]
*Populus* clone 717-1B4 (*Populus tremula* × *Populus alba*)	*PdGNC*	Carbon and nitrogen metabolism/TF	Reduced	KO	[133]
*Populus* clone NE-19 (*Populus nigra ×* (*Populus deltoides × P. nigra*))	*PdNF-YB21*	Flowering, growth, abiotic stress response/NF-Y TF	Reduced	KO	[134]
*Populus trichocarpa*	*PtrADA2b-3*	Chromatin modification/Histone acetyltransferase adaptor	Reduced	KO	[135]
*Solanum lycopersicum*	*SlALD1*	Stress responses/Pipecolic acid	Enhanced	KO	[136]
	*SlARF4*	Auxin signaling/Auxin response factor	Enhanced	KO	[137]
	*SlRR26*	Cytokinin pathway/Type-B Response Regulator	Enhanced	KO	[138]
	*SlSNAT2*	Negative regulation of rbcL/RUBISCO lysine acetylase	Enhanced	KO	[139]
	*SlLBD40*	Lateral root development/LBD TF	Reduced	KO	[140]
	*SlMAPK3*	Biotic and abiotic stress response/Mitogen-Activated Protein Kinase	Reduced	KO	[141]
	*SlNPR1*	Plant immunity/SA receptor	Reduced	KO	[142]
	*SP3C*	Anti-florigen/PEBP	Reduced	KO	[143]
*Solanum tuberosum*	*StFLORE*	Flowering/long non-coding RNA	Reduced	KD	[144]
*Triticum aestivum*	*TaSal1* *(6 homeologs)*	Monophosphate 3′-phosphoadenosine 5′phosphate (PAP) signaling	Enhanced	KO	[145]
	*TaCER1-6A*	Cuticle biosynthesis	Reduced	KO	[146]
	*TaIPT8*	Cytokinin biosynthesis/isopentenyltransferase	Reduced	KO	[147]
	*TaPYL1-1B*	Abscisic acid receptor	Reduced	KD	[148]
*Vitis vinifera*	*VvEPFL9-1*	Stomata formation	Enhanced	KO	[149]
*Zea mays*	*ARGOS8*	Negative regulator of ethylene responses	Enhanced	KU	[150]
	*ZmLBD5*	LBD Transcription factor	Enhanced	KO	[151]
	*ZmLRT*	lateral root Development/miR166a-encoding gene	Enhanced	KO	[152]
	*ZmPP84*	PP2C Phosphatase	Enhanced	KO	[153]
	*ZmSAG39*	Papain-like cysteine proteases	Enhanced	KO	[154]
	*ZmTCP14*	TCP Transcription factor	Enhanced	KO	[155]
	*ZmATHB-6*	Homeobox Transcription Factor	Reduced	KO	[156]
	*ZmEREB46*	Ethylene-responsive Transcription factor	Reduced	KO	[157]
	*ZmRBOHC*	NADPH oxidase	Reduced	KO	[158]
	*ZmRtn16*	Reticulon-like protein	Reduced	KO	[159]
	*ZmSRL5*	Cuticle biosynthesis	Reduced	KO	[160]
	*ZmSRO1d-S*	Oxidative and abiotic stress response	Reduced	KO	[158]

**Table 2:** Studies employing CRISPR/Cas on genes related to flooding stress. GA: Gibberellic Acid; ABA: Abscisic Acid; KO = Knockout.

Species	Target Locus	Pathway/Function	Effect on Tolerance	Result	Reference
*Oryza sativa*	*OsGF14h*	ABA and GA signaling/14-3-3 protein	Reduced	KO	[171]
	*SUB1A*	Ethylene-responsive transcription factor	Reduced	KO	[172]

## Data Availability

No new data were created or analyzed in this study. Data sharing is not applicable to this article.

## Supplementary Materials

The following supporting information can be downloaded at: https://www.mdpi.com/article/10.3390/plants12091892/s1, Table S1: Studies employing CRISPR/Cas on genes related to drought, flooding and waterlogging, salinity, heavy metal and toxic elements, and barrenness tolerance, extended.
